# Pd-C Catalytic Thin Films Prepared by Magnetron Sputtering for the Decomposition of Formic Acid

**DOI:** 10.3390/nano11092326

**Published:** 2021-09-07

**Authors:** Gisela Mariana Arzac, Asunción Fernández, Vanda Godinho, Dirk Hufschmidt, Maria Carmen Jiménez de Haro, Beatriz Medrán, Olga Montes

**Affiliations:** 1 Instituto de Ciencia de Materiales de Sevilla (CSIC-Univ. Sevilla), Avda. Américo Vespucio 49, 41092 Sevilla, Spain; godinho@icmse.csic.es (V.G.); dirk@icmse.csic.es (D.H.); cjimenez@icmse.csic.es (M.C.J.d.H.); medranbea@gmail.com (B.M.); olga@ciccartuja.es (O.M.); 2 Departamento de Química Inorgánica, Facultad de Química, Universidad de Sevilla, c/Profesor García González 1, 41012 Sevilla, Spain

**Keywords:** thin film, catalyst, magnetron sputtering, hydrogen, LOHC, formic acid, Pd-C

## Abstract

Formic acid is an advantageous liquid organic hydrogen carrier. It is relatively nontoxic and can be synthesized by the reaction of CO_2_ with sustainable hydrogen or by biomass decomposition. As an alternative to more widely studied powdery catalysts, supported Pd-C catalytic thin films with controlled nanostructure and compositions were newly prepared in this work by magnetron sputtering on structured supports and tested for the formic acid decomposition reaction. A two-magnetron configuration (carbon and tailored Pd-C targets) was used to achieve a reduction in Pd consumption and high catalyst surface roughness and dispersion by increasing the carbon content. Activity and durability tests were carried out for the gas phase formic acid decomposition reaction on SiC foam monoliths coated with the Pd-C films and the effects of column width, surface roughness and thermal pre-reduction time were investigated. Activity of 5.04 mol_H2_·g_Pd_^−1^·h^−1^ and 92% selectivity to the dehydrogenation reaction were achieved at 300 °C for the catalyst with a lower column width and higher carbon content and surface roughness. It was also found that deactivation occurs when Pd is sintered due to the elimination of carbon and/or the segregation and agglomeration of Pd upon cycling. Magnetron sputtering deposition appears as a promising and scalable route for the one-step preparation of Pd-C catalytic films by overcoming the different deposition characteristics of Pd and C with an appropriate experimental design.

## 1. Introduction

Our current energy paradigm is based on the extensive use of fossil fuels and is depleting natural resources and causing global warming. To reverse or at least attenuate this situation, it is necessary to move to a greener scenario. In this context, hydrogen (H_2_) appears to be an ideal energetic vector that can generate energy with water as the only product [1]. For the implementation of hydrogen as an alternative fuel, it is necessary to overcome numerous barriers, such as sustainable and low-cost production, transportation and storage [1,2,3,4,5]. The use of liquid organic hydrogen carriers (LOHCs) constitutes a very attractive strategy for hydrogen storage because of their high energy density and the possibility of using the existing infrastructure for fuel distribution [6,7,8]. Formic acid (HCOOH, FA) is a very interesting liquid hydrogen storage material because of its light weight and high hydrogen content (4.4 wt.%), low toxicity and recyclability [9,10,11,12,13,14]. It can be obtained by many methods, including biomass conversion, to achieve a carbon neutral cycle [15,16,17,18].

Formic acid generates hydrogen and carbon dioxide (CO_2_) according to dehydrogenation reaction (1) but it also generates carbon monoxide (CO) through its dehydration reaction (2). It is known that the pathway followed in the decomposition of formic acid (dehydrogenation or dehydration) is strongly dependent on the structure and composition of the catalyst surface, the formic acid concentration and the temperature [19].

HCOOH → H_2_ + CO_2_, (1)

HCOOH → H_2_O + CO, (2)

To date, many papers have reported the preparation of metal catalysts for the study of the decomposition of formic acid in the liquid phase (near room temperature) [20,21,22,23,24,25] as well as in the vapor phase [26,27,28]. For those applications in which FA decomposition is intended to be used as a hydrogen source to feed fuel cells, the room temperature reaction is preferred and the dehydration reaction is undesirable because carbon monoxide is poisonous for catalysts in cells. The study of catalysts for formic acid decomposition in the vapor phase is also of particular interest, as the dehydration reaction is favored at high temperatures [26]. Depending on the application, the formation of CO or a mixture of CO + H_2_ (syngas) may be desirable [29]. A recent investigation of the gas-phase decomposition of formic acid showed the potentiality of formic acid both as a CO and/or H_2_ carrier via selective dehydration and dehydrogenation reactions, respectively [29]. The study of the formic acid decomposition reaction in the vapor phase also permits us to achieve high conversion from catalysts such as thin films or single atom alloys, which in principle show low reaction rates at room temperature [28].

The most employed metallic catalysts for the dehydrogenation reaction are based on palladium because of its high activity (even at low temperatures), stability and selectivity [19,20,21,22,23,24,25,26,27,28,29,30]. In the literature, most Pd-based catalysts are prepared by wet chemistry, especially by impregnation. Proper supports are usually carbon-based, which improves the selectivity to reaction (1) [23,31]. Despite many reports of the successful preparation of Pd nanoparticles on carbonaceous supports, it is generally accepted that it is hard to find a correlation between preparation conditions and catalytic performance, and in some cases, the results are not easily reproducible [23,32,33,34]. As an alternative to wet chemical methods, physical vapor deposition (PVD) techniques offer advantages for catalyst preparation with controlled nanostructure and composition. One-step deposition can be performed on powdery and monolithic supports in a controllable, reproducible and industrially scalable procedure with little or no toxic byproduct generation. The importance of proper supports of active materials for efficient electrocatalytic reactions has been previously investigated in a variety of supports [35,36,37]. PVD techniques are advantageous due to their compatibility with varied support compositions. In particular, magnetron sputtering (MS) has attracted attention for the preparation of supported nanostructured electrocatalysts [38] and catalysts for hydrogen-involving reactions, such as the hydrolysis of sodium borohydride and the catalytic combustion of hydrogen [39,40,41,42].

In recent years, metal carbides have attracted attention as catalysts and electrocatalysts for many reactions [43]. Palladium carbides may be good candidates for selective formic acid decomposition, as suggested by theoretical calculations that indicate that the Pd–CO interaction is weakened when interstitial carbon is present [44]. Carbon-supported nonstoichiometric palladium carbide nanoparticles were reported previously in the study of electrocatalytic formic acid oxidation, but no studies were found for thermal FA decomposition [45].

In the present work, we explored for the first time, the MS deposition of nanostructured Pd-C thin films to be used as catalysts for the decomposition of formic acid in the vapor phase. Pd-C catalysts were deposited on monolithic SiC supports and the design of the experimental setup and deposition conditions were varied to improve surface roughness, dispersion and activity with low Pd consumption. The structure–composition–activity relationship, durability and effect of thermal pre-reduction time were studied for the formic acid dehydrogenation reaction.

## 2. Materials and Methods

### 2.1. Preparation of Samples

Three Pd-C thin film catalysts were deposited by magnetron sputtering on SiC and silicon wafer supports. The deposition chamber was equipped with two magnetrons (2” diameter) from the AJA International Inc. Company. In all preparations, Ar was used as the process gas (10^−2^ mbar working pressure) and a bias voltage (150–220 V) was employed to increase the adhesion of the growing films. The substrates were placed on a rotary (and biased) sample-holder to ensure homogeneous deposition.

**Figure 1:**
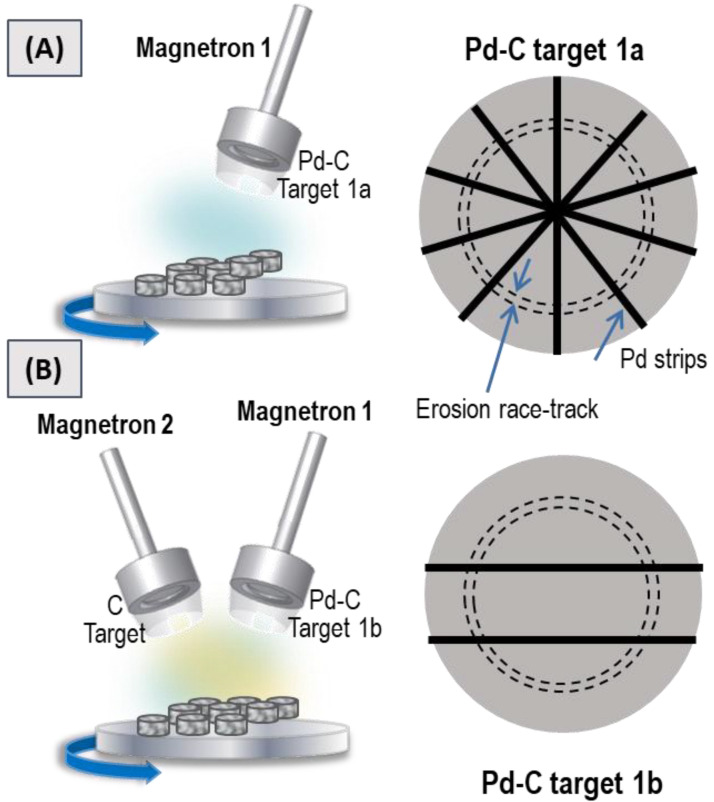
Scheme of the sputtering chamber and homemade Pd-C targets employed to prepare (**A**) 7C and 12C samples, and (**B**) the 65C sample.

Minimizing Pd consumption during co-deposition of Pd and C was our first objective. To achieve this objective, we employed a homemade target composed of a commercial C target with Pd strips in different amounts and configuration. This methodology was inspired by our previous work [41] in which we presented a cost-effective method for the fabrication of Pt-based catalytic coatings by MS. In that case, a tailored target was fabricated with a conventional Cu target on which four Pt strips were radially distributed. In the present work, we intended to employ the same strategy by using a conventional carbon target with Pd strips. Therefore, we first tested the single magnetron configuration shown in Figure 1A. According to the conditions summarized in Table 1, two samples were first prepared by operating a single magnetron head furnished with a homemade target (magnetron 1 and target 1a in Figure 1A) at different power levels. This target consisted of a commercial carbon target (Neyco 99.99% pure) with five Pd strips (Sigma Aldrich wire, diameter of 1.0 mm, 99.9% trace metal basis) arranged according to Figure 1A. The power applied to the “Pd + C” target was 70 and 200 W. The fabricated samples were named 7C and 12C, respectively, according to their carbon content, as it will be discussed in the Section 3.

As C has higher sublimation energy than Pd, much lower sputtering yields are expected for carbon [46]. In order to increase the C content, a third sample was prepared by using a two-magnetron configuration: one head (magnetron 2, Figure 1B) operated with a single carbon target (Kurt J. Lesker 99.99% pure) and the other head (magnetron 1, Figure 1B) operated with a homemade target consisting of the commercial carbon target (Neyco 99.99% pure) with only two Pd strips (Sigma Aldrich wire, diameter of 1.0 mm, 99.9% trace metal basis) arranged in a parallel geometry (target 1b). The power applied to the carbon target was 500 W and 200 W was applied to the homemade “Pd + C” target. The third sample was named 65C according to the carbon content and it will be discussed in the Results section. For the catalytic studies and/or characterization, all the coatings were directly deposited under the conditions described above onto two substrates: (i) commercial alumina-bonded SiC monolith foam (Vukopor S100 Lanik, 16 mm diameter, 3 mm thickness, 100 pores/inch) which was washed in an ultrasonic bath successively with distilled water and ethanol/acetone (1:1) and then dried in a vacuum at 110 °C overnight; and (ii) pieces of a Si (100) wafer (from A.C. M) cleaned with acetone and dried with a nitrogen flow.

Alternative methodologies have been previously reported to fabricate catalytic Pd-C films by pulsed laser deposition [47] and by a two-step PVD/CVD (physical/chemical vapor deposition) process [48]. The methodology presented here is simpler (one step), is scalable, and ensures the low consumption of the noble metal and recycling of target materials, and to the best of our knowledge, was newly tested with Pd-C catalysts for the formic acid decomposition reaction.

### 2.2. Characterization

Scanning electron microscopy (SEM) analyses were performed on silicon and SiC foam-supported films using a Hitachi S4800 high-resolution SEM-FEG (field emission gun) microscope operated at 5 keV for imaging and at 10 keV for compositional analysis. The microscope was equipped with a Bruker X-Flash 4010 energy dispersive X-ray (EDX) detector to study the catalyst composition. Samples deposited on Si substrates were cleaved and observed without metallization in cross-sectional views.

X-ray diffraction (XRD) measurements were performed on the Si- and SiC-supported films using a Siemens D5000 diffractometer with Cu Kα radiation in a Bragg-Brentano configuration. Data were collected in the 2θ range of 10–90 degrees.

For transmission electron microscopy (TEM) studies, SiC-supported Pd-C films were ground in a mortar to obtain a powder. Cross-sectional transmission electron microscopy (XTEM) specimens were prepared from thin films deposited on Si substrates in the conventional way by mechanical polishing followed by Ar+ ion milling to achieve electron transparency. Measurements were performed by employing a Jeol 2100Plus microscope operated at 200 kV.

X-ray photoelectron spectroscopy (XPS) spectra of SiC-supported films were recorded with a SPECS electron spectrometer equipped with a PHOIBOS 150 hemispherical analyzer using Al Kα radiation with 35 eV pass energy and normal emission take-off angle. The spectra were calibrated with the C 1s signal at 284.6 eV attributed to adventitious carbon. Data were analyzed and deconvoluted when necessary, by using the CASA XPS^®^ software. Sensitivity factors were extracted from a previous publication [49].

To quantify the Pd content the samples deposited on SiC discs were acid digested in a dedicated Milestone-UltraWAVE digestor. After adequate dilution, elemental analyses were done by inductively coupled plasma-atomic emission spectroscopy (ICP-AES) at the CITIUS laboratories (University of Seville) in a SpectroBLUE spectrometer.

### 2.3. Catalytic Tests

The formic acid decomposition reaction at the solid–gas interface was studied using Pd-C thin films supported on SiC monoliths. Samples were loaded in a tubular quartz reactor (20 mm diameter, 500 mm length) and heated from the outside by using a Hobersal TR0 electric furnace. A thermocouple was in contact with the monolithic catalyst. Formic acid was introduced in the gas phase by using N_2_ as the carrier gas and a controlled evaporation mixer (CEM) unit from Bronkhorst to control the formic acid/carrier gas composition and the temperature, which was set to 100 °C. The carrier gas flow rate was 193.5 mL·min^−1^, and the FA flow rate was 0.8 g·h^−1^. This results in a 3.2 mol% or 1.5 v/v composition of FA in N_2_ and a space gas velocity of 0.0165 m·s^−1^. To avoid formic acid condensation, the pipes were maintained at 60 °C during the experiments. The exhaust gas was analyzed with a proper gas chromatography (GC) system consisting of two gas chromatographs (HP 7890B) connected in line. The first chromatograph was equipped with a CPSIL^®^ capillary column and a thermal conductivity detector (TCD) and used to monitor the formic acid concentration. The second chromatograph was equipped with an Agilent CARBOPLOT^®^ capillary column and both a TCD and flame ionization detector (FID) and was used to monitor the CO/CO_2_ concentration ratio with a detection limit below 100 ppm. Conversion was tested as a function of the temperature in cooling mode. Before activity measurements, samples were pre-reduced with a H_2_ (50 mL·min^−1^) and N_2_ (100 mL·min^−1^) mixture at 400 °C for 1.5 h. A longer pre-reduction time of 3 h was also tested but resulted in a further decrease in the activity. After pre-reduction, the reactor was cooled to 360 °C under N_2_ flow, and then, it was fed the FA/N_2_ mixture. The reactor was slowly cooled, and conversion was measured as a function of temperature (once the steady state was achieved at each temperature). FA % conversion was calculated by using equation 3, where AREA FA(T) represents the area of the FA peak at temperature T and AREA FA (0) represents the area of FA at 0% conversion at the end of each experiment.

(3) $$\mathrm{FA}\;\%\;\mathrm{conversion}=100\times \left(1-\frac{\mathrm{AREA}\;\mathrm{FA}\left(T\right)}{\mathrm{AREA}\;\mathrm{FA}\;\left(0\right)}\right)$$

CO_2_% selectivity was calculated as shown in equation 4, where AREA CO_2_ and AREA CO correspond to the peak areas of CO_2_ and CO, respectively.

(4) $${\mathrm{CO}\;}_{2}\%\;\mathrm{selectivity}=100\times \left(\frac{{\mathrm{AREA}\;\mathrm{CO}}_{2}}{\mathrm{AREA}\;\mathrm{CO}+{\mathrm{AREA}\;\mathrm{CO}}_{2}}\right)$$

Activity was expressed in units of turnover frequency (TOF) in mol_H2_·mol_Pd_^−1^·h^−1^, and in units of mol_H2_·g_Pd_^−1^·h^−1^. The moles of hydrogen produced were calculated by considering the FA feed rate, the stoichiometry of the reaction and the conversion and selectivity to CO_2_ at a certain temperature. The moles of Pd were calculated by ICP measurements.

(5) $$\mathrm{TOF}=\frac{n_{\mathrm{mol}\mathrm{H2}}}{n_{\mathrm{molPd}}.h}$$

(6) $$\mathrm{Activity}=\frac{n_{\mathrm{mol}\mathrm{H2}}}{m_{\mathrm{Pd}}.h}$$

## 3. Results and Discussion

### 3.1. Catalysts Characterization

**Figure 2:**
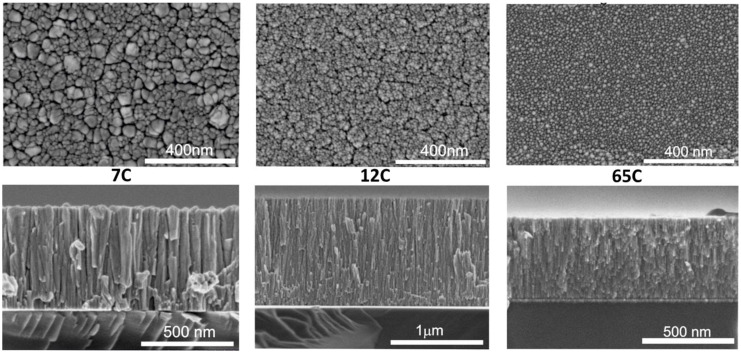
Top- and cross-sectional SEM images of Si-supported Pd-C coatings with different compositions.

Samples were first studied by SEM and EDX analysis to find the expected correlation between deposition conditions, composition and microstructure. Samples were therefore named by considering their EDX determined carbon content with values of 7, 12 and 65 at.%, as summarized in Table 2. Figure 2 shows the SEM images (top and cross-section views) for the Si-supported films. Samples show a columnar microstructure with inter- and intra-columnar porosity. By observing the top-view images it can be concluded that the column width decreases with carbon content, which correlates with a higher surface roughness and catalyst dispersion. Average column size is also included in Table 2. The microstructure of MS-deposited films is strongly influenced by the atomic surface mobility on the growing film [46]. Carbon has a much higher melting point than palladium, which favors ballistic deposition and smaller columns with intercolumnar voids. The sample with the highest amount of carbon (65C) is shown to be the most disperse, with a smaller column width. A similar inverse correlation between surface roughness and column width was found in our previous work on Co catalysts for the hydrolysis of sodium borohydride [39]. The same correlation was found for the Pd-C films supported on SiC, as shown in Figure S1. This permits us to combine the results obtained by different techniques for films supported on different substrates, according to the requirements of each characterization method.

**Figure 3:**
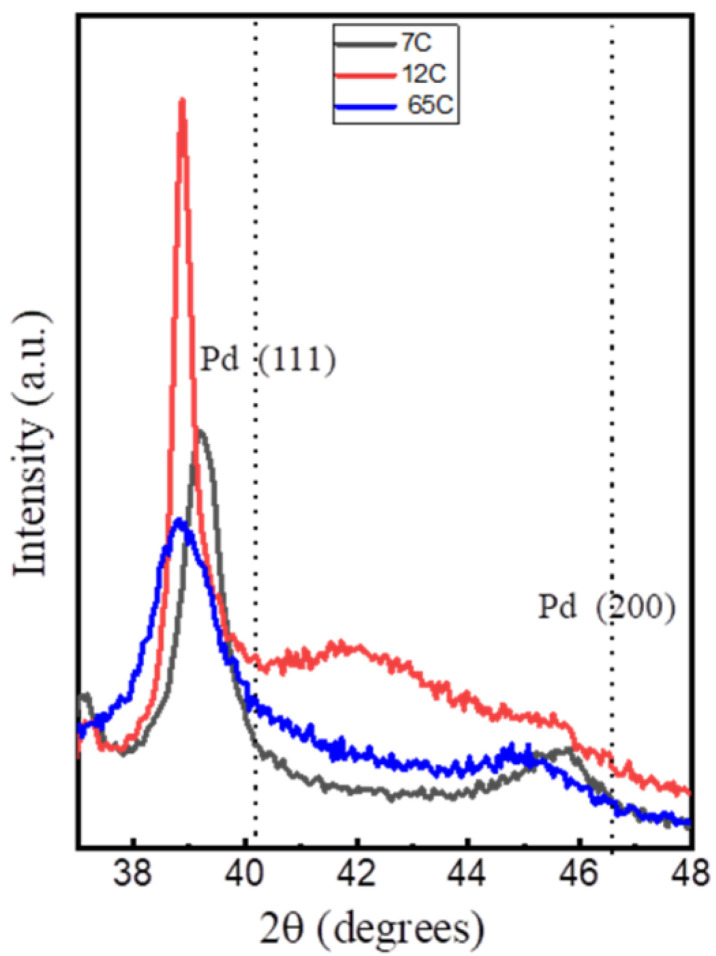
XRD patterns of the Si-supported Pd-C coatings with different compositions. Dotted lines indicate the position of pure Pd peaks.

**Figure 4:**
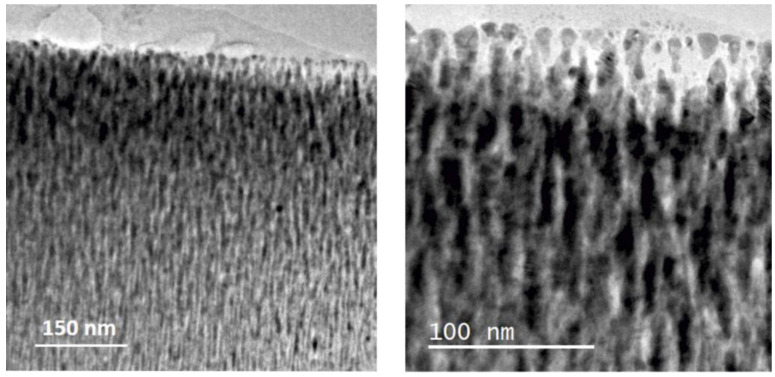
Cross-sectional TEM images of the 65C sample.

The XRD patterns for the Si-supported Pd-C films are shown in Figure 3. The Pd (111) peak is marked on the pattern for reference. The samples show a negative shift in the position of these reflections with respect to pure metallic Pd, which can be attributed to the presence of interstitial carbon [50]. The peak positions correlate with those reported for PdC_x_ phases [50], with x < 0.13 for the 7C and x = 0.13–0.15 for the 12C and 65C samples. The 7C and 12C samples show broader and sharper peaks, respectively, as expected from the low and higher power used during the MS deposition. Therefore, the 12C sample contains more and larger crystalline domains derived from the Scherrer equation (see data in Table 2). The 65C sample shows the smallest crystal size (10.2 nm), which is consistent with the lower column width and higher surface roughness observed by SEM and the high C content measured by EDX analysis. This sample was also studied by TEM, as shown in Figure 4, showing the high degree of inter- and intracolumnar porosity, which significantly increases the active surface roughness and area. As complementary information, we have included SEM top-view images at low magnification (Figure S2) for the bare and 65C catalyst-coated SiC foam support. The conformal deposition shows the improved contact surface area for catalysis through the monolithic foam supports. The methodology presented here may also be of interest in regard to highly porous supports (e.g., porous carbons) for catalytic and electrocatalytic [51] applications.

**Figure 5:**
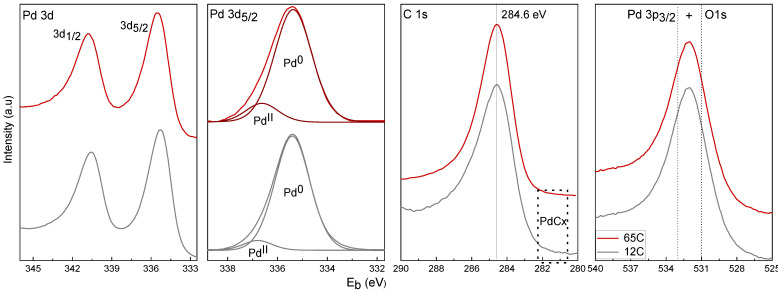
XPS spectra of the 12C and 65C samples as deposited in SiC foam.

For the XPS surface analysis, we have selected the 12C and 65C samples (deposited on SiC discs) as representative of the low and high carbon content range, respectively. The results for selected regions of surface-detected Pd, C and O elements are depicted in Figure 5. Quantification of the Pd3d and C1s peaks with the adequate sensitivity factors lead to the Pd/C atomic % ratios. Data are included in Table 2. The relative atomic amount of surface C is significantly higher for 65C (88%) than for 12C (40%) despite the obvious presence of adventitious surface carbon.

The study of the Pd 3d_5/2_ level shows the presence of a broad signal at 335.5 eV, consistent with the presence of Pd in the reduced state. Deconvolution was carried out to evaluate the contributions of the individual oxidation states. Peaks were adjusted by using the 3d_5/2_ signal with the following constraints according to the literature [50,52,53,54]: the peak position of the Pd^0^ signal was set in the range of 354.9–355.5 eV, the PdC_x_ signal was set at +0.6 eV in respect to the Pd^0^ interval, and the Pd^II^ signal was at +1.6 eV in respect to the Pd^0^ interval. Individual components corresponding to Pd^0^ and Pd^II^ were plotted after background removal. No components corresponding to PdC_x_ were detected, which is in accordance with the absence of signal of carbidic carbon in the range of 281–282 eV in the C 1s level. The presence of surface adventitious carbon makes the surface detection of the PdC_x_ difficult, which on the other hand has been well characterized by the bulk XRD analysis. The percentages of Pd^0^ and Pd^II^ were 93% and 7%, respectively, for the 12C sample, and 87% and 13%, respectively, for the 65C sample. The higher degree of surface oxidation of the high Pd-C sample is in agreement with its lower column and higher surface roughness. The presence of oxygen is confirmed by the study of the O 1s level, which shows the signal of PdO at 531 eV, which overlaps the Pd 3p_3/2_ peak at 533 eV [50,52,53,54]. Table 2 also includes the amount of Pd in the films (determined by digestion and ICP-AES analysis) per unit of geometrical area (cm^2^) of the porous SiC disks. These values are comparable in the three samples if the film thicknesses are considered. The above results demonstrate that the increase in carbon content from the 7C to 12C and 65C samples controls the Pd-phase dispersion, as required.

### 3.2. Catalytic Activity and Durability Tests

**Figure 6:**
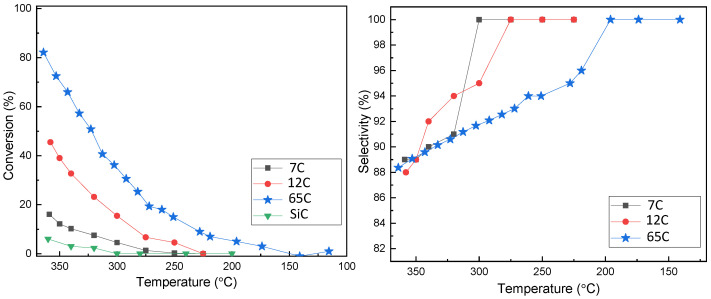
Conversion and selectivity as a function of temperature for Pd-C thin films with different compositions.

Activity measurements were performed on the three Pd-C samples with different compositions, and the results are shown in Figure 6. The activity of bare SiC is also shown for reference. The three samples showed measurable activity for the decomposition of formic acid in the studied temperature range. As the amounts of Pd in the tested samples are not strictly equal (mainly due to different thicknesses), the curves should be compared carefully. For a more precise comparison, TOF (mol_H2_·mol_Pd_^−1^·h^−1^) and activities (mol_H2_·g_Pd_^−1^·h^−1^) were calculated, and the results are shown in Table 2. As an approximation, the whole amount of Pd obtained by ICP was considered for these calculations. Metal dispersion or active accessible metallic surface area were difficult to measure due to the low mass ratio of the thin film catalyst to the support material. The activity trend is in good accordance with the higher catalyst dispersion and roughness attributed to higher carbon content and lower column width. The activity of 65C catalyst is approximately eight times higher than that of 7C (534 vs. 66 mol_H2_·mol_Pd_^−1^·h^−1^). The strategy of decreasing the column width and increasing surface roughness by adding carbon together with the formation of the PdC_x_ phase (detected by XRD) has been proven to be effective in increasing the activity.

Selectivity to CO_2_ was also studied and is shown in the same figure. It is generally accepted that higher temperatures decrease the selectivity to CO_2_, favoring the dehydration reaction [19,26]. Both the 7C and 12C samples produced 100% CO_2_ at temperatures below 270 °C. However, the 65C sample showed less selectivity, being 100% selective at temperatures below 200 °C. The preference for the dehydration vs. dehydrogenation pathways has been correlated to the acidic vs. basic character of the catalyst’s surface [29]. This can be affected by modification of the electronic state and adsorption properties of Pd by the incorporation of carbon in Pd-C solid-solutions [44,45]. When we compared the 7C and 65C samples, which are of similar film thickness and crystallite size, a larger amount of incorporated carbon reduced the CO_2_ selectivity, as suggested by previous works on FA electro-oxidation [45]. Arrhenius plots are shown in Figure S3, and apparent activation energies (AAEs) were calculated (Table 2). The 65C sample exhibits the lowest activation energy, consistent with its highest activity.

To benchmark the activity of the 65C sample, it is necessary to compare it with catalysts shown in the literature and tested in identical conditions. This constitutes a difficult task, and for this reason, we prepared 5 nm Pd particles supported on carbon (Norit ^®^) by incipient wetness impregnation for comparison purposes. Figure S4 shows the activity test and the TEM images. The TOF at 300 °C was 4000 h^−1^. In comparison with the 65C catalyst (534 h^−1^), and with other catalysts tested at near room temperature [10,11,12,13,14,20,21,22,23,24,25], it is evident that the activity should be improved in further works. Additional research should be conducted to increase the carbon content, surface area and porosity of the films. The proposed strategies are oblique and glancing angle deposition, or even employing MS for the preparation of Pd-C samples in the form of nanoparticles [42]. The development of tools for quantification of the surface area and metallic phase dispersion is also highly desirable, as this is not as straightforward in thin films as it is for powdery materials, as discussed in our recent review paper [42].

**Figure 7:**
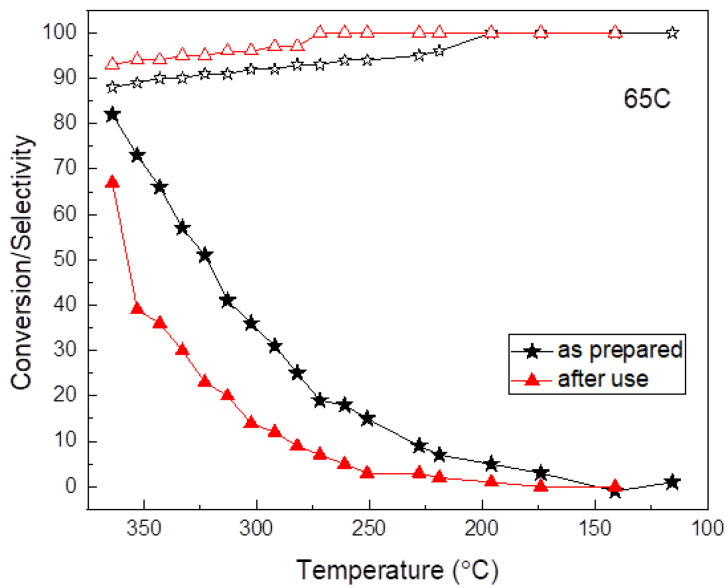
Durability test of the 65C thin film. Conversion (full symbols) and selectivity (open symbols) as a function of temperature.

**Figure 8:**
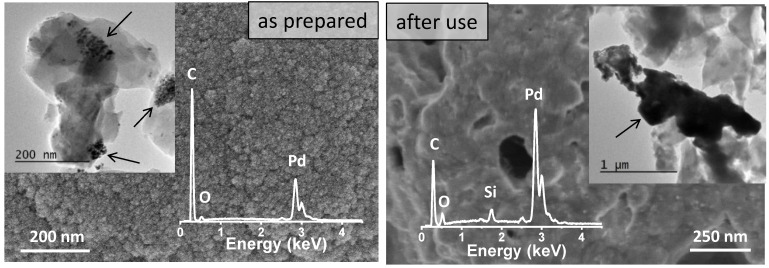
SEM and TEM images and SEM-EDX analysis showing the effect of catalytic tests on the 65C thin film. Catalytic films are indicated by arrows in the TEM images.

The durability of the 65C sample was tested upon cycling. Figure 7 shows a comparison of the conversion and selectivity as a function of temperature for this catalyst before and after use. Clearly, the sample deactivates upon use and the reasons were investigated. SEM, SEM-EDX and TEM characterization were done first and the results are shown in Figure 8. Before use, the as-prepared sample showed high surface roughness by SEM, while after use the formation of aggregates and a sort of thick film was observed at the surface. EDX compositional data indicated there were larger amounts of Pd in comparison to C in the deactivated sample (additional Si signal comes from the SiC substrate). The comparison of the TEM images shown in Figure 8 suggests that Pd aggregation should be the reason for deactivation.

**Figure 9:**
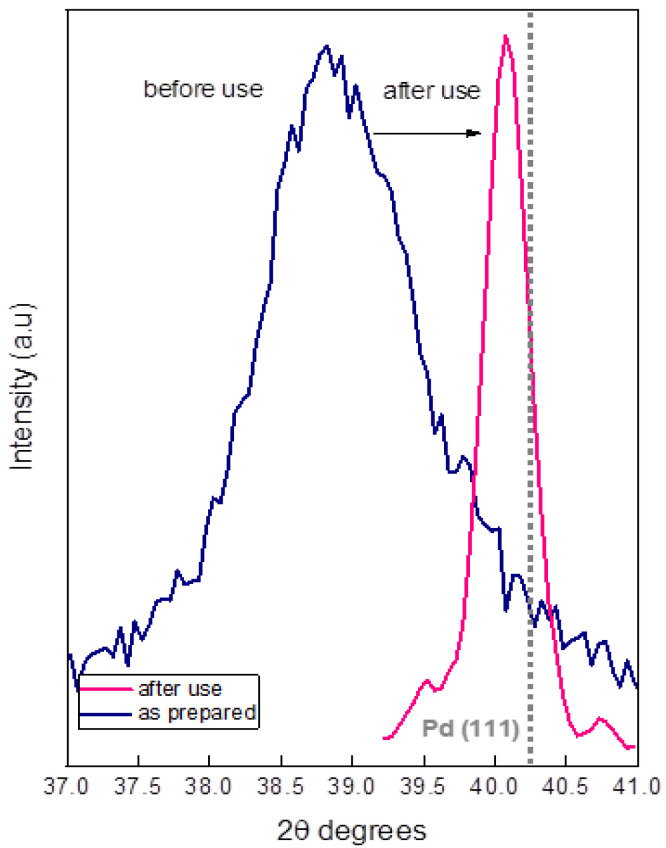
X-ray diffractograms of the as-prepared and used 65C catalyst.

To better understand the results in Figure 8, we studied a powdery sample obtained from the top layer of SiC monoliths by XRD after the first catalytic test. In Figure 9, the as-prepared 65C sample was compared to the sample after use. The position of the PdC_x_/Pd (111) reflections shifted to a higher Pd content in the case of the used sample, indicating the formation of metallic Pd. Calculation of the crystal size by using the Scherrer equation confirms the aggregation process that was inferred by electron microscopy. The comparison of the crystal size of the as-prepared 65C sample (10.2 nm) with the crystal size of the sample after use (58 nm) was conclusive: upon use of the catalytic films at high temperatures, the active Pd phase aggregates and thus decreases the active surface area, causing deactivation. This aggregation may be favored by Pd segregation and/or the loss of carbon during catalytic tests, which favors the ripening of Pd atoms to form an essentially metallic catalyst surface [55]. Some authors proposed that the adsorption of CO on Pd atoms facilitates their movement and particle growth in Pd/Ceria catalysts for the water–gas shift reaction [56]. In our 65C sample, such a mechanism could also occur since we detected the formation of CO at higher temperatures. The improvement in selectivity with cycling is explained by the loss of carbon and the formation of the metallic phase.

A similar effect should occur with pre-reduction time. When we increased this from 1.5 h to 3 h, the activity significantly decreased, showing aggregation in the TEM images (Figure S5). This occurs due to the synergism between the high temperatures, the loss of carbon during reduction and the Pd segregation.

## 4. Conclusions

In the present work, we prepared, for the first time, a Pd-C thin-film catalyst by magnetron sputtering for the decomposition of formic acid in the vapor phase. In spite of the very low sputtering yield of carbon in comparison to palladium, we were able to control the nanostructure and composition by the proper design of deposition conditions using homemade palladium and carbon composite targets. The thin films were columnar, and we found a correlation between the carbon content, column width and surface roughness. The increase in carbon content led to a decrease in column width with inter- and intracolumnar porosity, and then to an increase in surface roughness, catalyst dispersion and higher activity. An active PdC_x_ phase has also been identified, which could explain the increase in activity and the decrease in selectivity. We studied the durability upon cycling of the 65C catalyst with the highest dispersion, and we found that the sample deactivated upon use. The deactivation mechanism was investigated, and we found that the Pd atoms sintered due to segregation and/or to the elimination of carbon upon use. The Pd-C films are thus unstable for operation at high temperatures.

In sum, this work shows the potentiality of magnetron sputtering for the preparation of palladium-carbon-based catalysts for the gas phase decomposition of formic acid. The one-step, low Pd consumption and scalable deposition of active films on structured supports was demonstrated. In order to increase activity at lower temperatures, where good selectivity is also expected, future work is foreseen to increase the surface roughness (higher carbon content), area and porosity. The control of microstructure and composition offered by magnetron sputtering, from pure Pd targets to the here proposed co-sputtering procedure, in a wide range of structured supports (including porous carbons) will strongly contribute to the development of both the catalytic decomposition and the electro-catalytic oxidation [51,57] of formic acid.

## Figures and Tables

**Table 1:** MS deposition conditions for catalyst preparation.

Catalyst	Magnetron 1		DC Power (W) Magnetron 1	Magnetron 2 Target 2 C	DC Power (W) Magnetron 2
	Target-1a 5 Pd Strips/C	Target-1b 2 Pd Strips/C			
7C	Yes		70		
12C	Yes		200		
65C		Yes	200	Yes	500

**Table 2:** Catalysts’ characterization.

Sample	Bulk at% Ratio Pd/C (EDX)		Surface at % Ratio Pd/C (XPS)		ICP Pd Content (mg·cm^−2^)	SEM Measurements		XRD Measurements		Activity Measurements			
	% Pd	%C	% Pd	%C		Thick-ness (nm)	Column Size (nm)	Peak Position (degrees)	Crystal Size (nm)	TOF (a) 300 °C	Activity (b) 300 °C	Selectivity to CO_2_ (%) 300 °C	AAE (kJ·mol^−1^)
7C	93	7	-	-	0.565	549 ± 15	64 ± 28	39.21	13.8	66	0.60	100	61 ± 4
12C	88	12	60	40	1.15	1168 ± 6	51 ± 27	38.85	32.5	105	1.59	95	55 ± 1
65C	35	65	12	88	0.492	435 ± 2	9 ± 4	38.97	10.2	534	5.04	92	42 ± 1

(a) (mol_H2_·mol_Pd_^−1^·h^−1^); (b) (mol_H2_·g_Pd_^−1^·h^−1^).

## Data Availability

The data presented in this study are available on request from the corresponding authors.

## Supplementary Materials

The following are available online at https://www.mdpi.com/article/10.3390/nano11092326/s1, Figure S1: Top SEM images for the SiC supported Pd-C coatings with different composition title, Figure S2: Experimental proton EBS spectrum from the High Pd-C film supported on silicon, Figure S3: Arrhenius plots for the Pd-C coatings with different composition, Figure S4: Activity and selectivity as a function of temperature and particle size of a 0.5 wt.% Pd catalyst supported on carbon (Norit ®), Figure S5: Effect of the pre-reduction time in the activity and after use microstructure (TEM analysis) of the High Pd-C thin film.
