# Supplementary material for: Pd-C Catalytic Thin Films Prepared by Magnetron Sputtering for the Decomposition of Formic Acid
Source: Nanomaterials (Basel). 2021 Sep 7;11(9):2326. doi: 10.3390/nano11092326 (PMC8466502; doi:10.3390/nano11092326)
Supplement: Supplementary file 1 [file nanomaterials-11-02326-s001.zip › nanomaterials-1358305-supplementary.pdf]

nanomaterials

Article

Pd-C Catalytic Thin Films Prepared by Magnetron Sputtering for the Decomposition of Formic Acid

Gisela Mariana Arzac [1,2,*], Asunción Fernández [1,*], Vanda Godinho [1], Dirk Hufschmidt [1], Maria Carmen Jiménez de Haro [1], Beatriz Medrán [1] and Olga Montes [1]

[1] Instituto de Ciencia de Materiales de Sevilla (CSIC-Univ. Sevilla), Avda. Américo Vespucio 49, 41092 Sevilla, Spain; godinho@icmse.csic.es (V.G.); dirk@icmse.csic.es (D.H.); cjimenez@icmse.csic.es (M.C.J.d.H.); medranbea@gmail.com (B.M.); olga@ciccartuja.es (O.M.)

[2] Departamento de Química Inorgánica, Facultad de Química, Universidad de Sevilla, c/Profesor García González 1, 41012 Sevilla, Spain

* Correspondence: gisela@icmse.csic.es (G.M.A.); asuncion@icmse.csic.es (A.F.)

Citation: Arzac, G.M.; Fernández, A.; Godinho, V.; Hufschmidt, D.; Jiménez de Haro, M.C.; Medrán, B.; Montes, O. Pd-C Catalytic Thin Films Prepared by Magnetron Sputtering for the Decomposition of Formic Acid. *Nanomaterials* **2021**, *11*, 2326. https://doi.org/10.3390/nano11092326

Academic Editor: Giuseppe Cappelletti

Received: 11 August 2021
Accepted: 3 September 2021
Published: 7 September 2021

Publisher's Note: MDPI stays neutral with regard to jurisdictional claims in published maps and institutional affiliations.

Copyright: © 2021 by the authors. Licensee MDPI, Basel, Switzerland. This article is an open access article distributed under the terms and conditions of the Creative Commons Attribution (CC BY) license (http://creativecommons.org/licenses/by/4.0/).

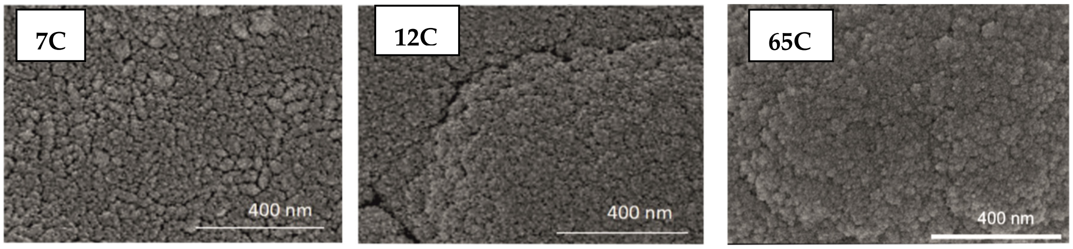

Figure S1. Top SEM images for the SiC supported Pd-C coatings with different composition.

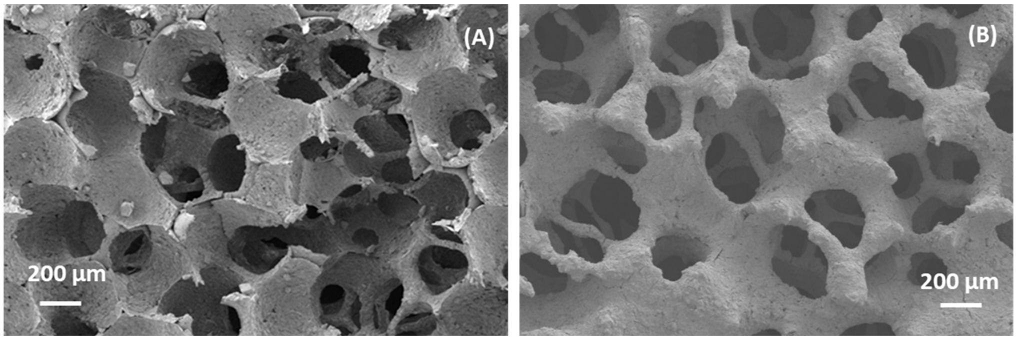

Figure S2. SEM images at low magnification for the SiC foam support: Before (**A**) and after (**B**) the Pd-C catalyst (65C) was deposited.

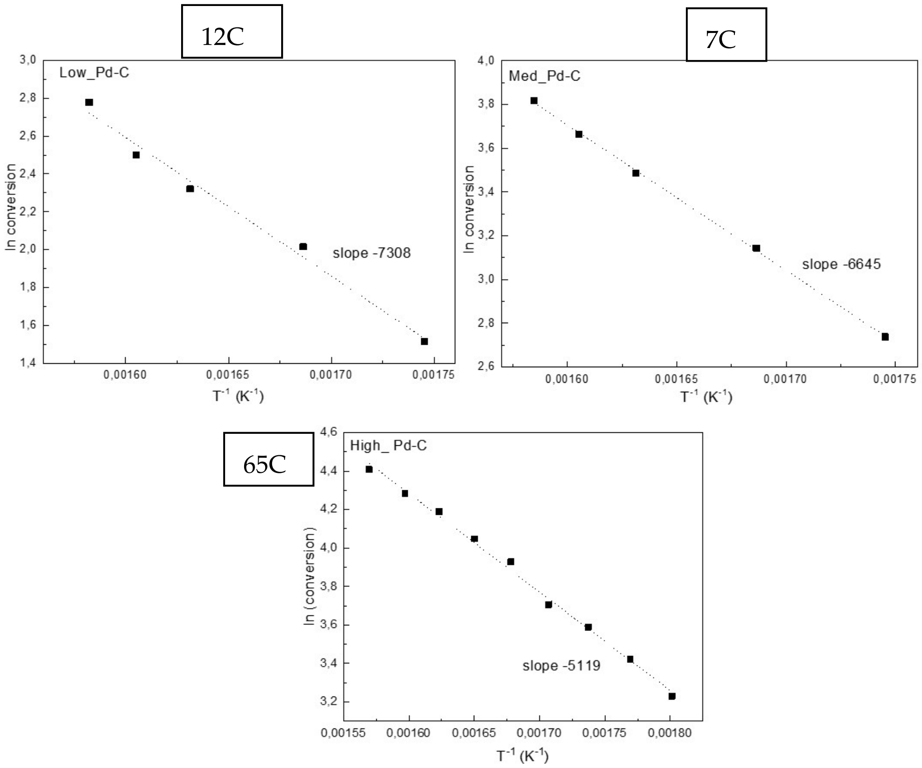

Figure S3. Arrhenius plots for the Pd-C coatings with different composition.

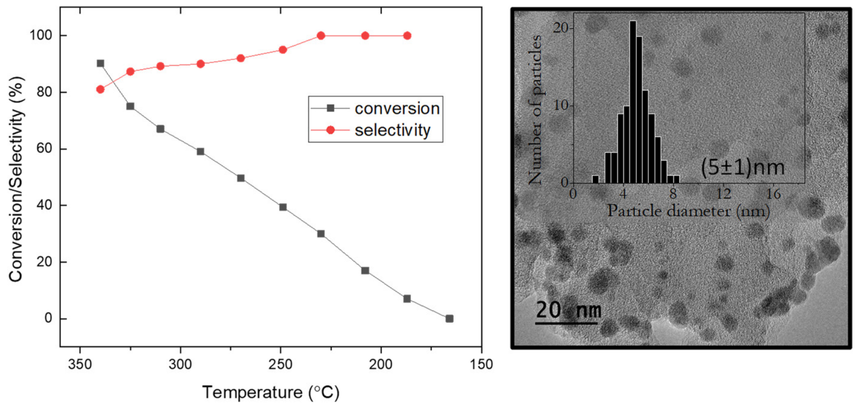

Figure S4. Activity and selectivity as a function of temperature and particle size of a 0.5 wt.% Pd catalyst supported on carbon (Norit ®). The amount of catalyst tested was 50mg. The conditions of the test were identical as the ones reported in the present work, and the catalyst was diluted by using quartz beads. The catalyst was prepared by incipient wetness impregnation. The support was previously activated by a treatement with 10wt% HCl solution in order to generate acid sites for better anchorage of Pd particles. The pore volume impregnation was conducted at Ph = 1. 5. The Pd precursor was Na_2PdCl_4. After impregnation the catalyst was dried in a vacuum oven at 40ºC overnight. Sample was pre-reduced before testing as done in the whole present work.

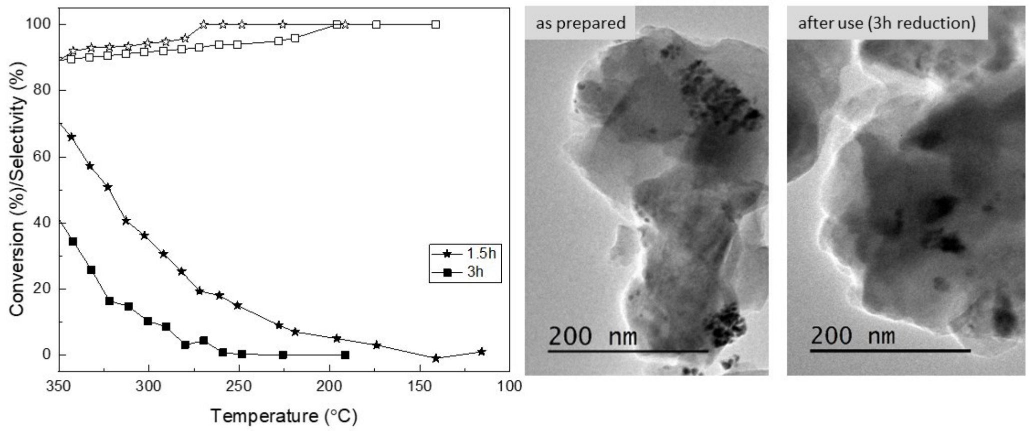

Figure S5. Effect of the pre-reduction time in the activity and after use microstructure (TEM analysis) of the 65C sample.
